# From Disease‐Specific Models to Broad Clinical Utility: A Perspective on AI Hybrid Ensemble Frameworks

**DOI:** 10.1002/ggn2.202500053

**Published:** 2026-03-16

**Authors:** Haonan Zhang, Ge Zhang, Chaoyang Yu, Ruhao Wu, Shiqian Zhang, Xufeng Huang, Yingxue Yuan, Yaxin Chen, Shaotong Pei, Ge Zhang

**Affiliations:** ^1^ Department of Thyroid Surgery The First Affiliated Hospital of Zhengzhou University Zhengzhou Henan China; ^2^ Department of Gastroenterology The First Affiliated Hospital of Zhengzhou University Zhengzhou Henan China; ^3^ The First Affiliated Hospital of Zhengzhou University Zhengzhou Henan China; ^4^ Department of Colorectal Surgery Tianjin Union Medical Center and The First Affiliated Hospital of Nankai University Medical College of Nankai University Tianjin China; ^5^ Department of Data Visualization Faculty of Informatics University of Debrecen Debrecen Hungary; ^6^ The First Affiliated Hospital of Zhengzhou University Zhengzhou University Zhengzhou Henan China; ^7^ School of Clinical Medicine Zhengzhou University Zhengzhou Henan China

**Keywords:** bioinformatics, computional biology

## Abstract

Artificial intelligence (AI) has advanced predictive modeling in medicine, yet many models remain disease‐specific and difficult to generalize across clinical settings. Key challenges include the trade‐off between interpretability and accuracy, reliance on single algorithms, limited external validation, and biased feature importance estimation. In this Perspective, we discuss how methodological advances in computational sciences, including automated machine learning (AutoML) and neural architecture search (NAS), reveal a gap between automated hybrid systems and current clinical modeling practices. To address these challenges, we outline a principled artificial intelligence hybrid ensemble framework based on three design principles: integration of diverse learners, consensus‐driven validation across independent cohorts, and transparent feature attribution using Shapley Additive exPlanations (SHAP). This framework emphasizes methodological robustness, interpretability, and cross‐disease applicability to support the translation of artificial intelligence models into clinical practice.

## Graphical abstract

An artificial intelligence (AI) hybrid ensemble framework integrates multiple learners with recursive feature elimination and consensus evaluation, achieving robust and interpretable predictions. By balancing accuracy, interpretability, and complexity, this approach addresses high‐dimensional challenges and enhances clinical utility, offering a generalizable paradigm for reliable prognostic modeling across diverse medical domains.

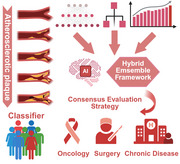

## 1 Introduction

Artificial intelligence (AI) is increasingly being applied in predictive medicine, offering new opportunities for modeling complex clinical phenomena. While initial successes have been demonstrated in disease‐specific contexts, a persistent gap remains between narrowly optimized, high‐accuracy models and predictive tools with broad clinical utility. Many existing approaches are tailored to specific datasets or clinical questions, often at the expense of interpretability, robustness across populations, and integration into heterogeneous clinical workflows. These limitations highlight the need for methodological frameworks that extend beyond single‐disease applications and emphasize generalizable principles for reliable clinical prediction.

In this Perspective, we argue that the next phase of clinical AI development should prioritize methodological generalizability alongside predictive performance. Rather than introducing a new disease‐specific model, we offer a structured viewpoint on AI hybrid ensemble frameworks, emphasizing design principles that balance accuracy with interpretability, mitigate overfitting through consensus‐driven validation, and enhance transparency via explainable methods. Our aim is to reframe clinical AI development toward methodologies that are not only performant but also robust, interpretable, and suitable for real‐world clinical deployment [1, 2, 3].

## 2 Current State and Critical Gaps: Beyond Technical Performance

The past few years have witnessed significant progress in data‐driven modeling across clinical domains, from oncology to perioperative estimation. Foundation models, which combine machine learning algorithms, have gained traction for their ability to improve predictive stability and accuracy. However, several methodological and translational gaps remain largely unaddressed:

1. The Interpretability‐Accuracy Trade‐off: Many high‐performing models operate as “black boxes”, offering limited insight into the rationale behind predictions, which erodes clinician trust and hinders adoption [4].
2. Over‐reliance on single algorithms: Dependence on a single modeling approach may introduce generalization error and reduce robustness across datasets with varying characteristics [5].
3. Validation Silos and Overfitting: Models are frequently developed and validated on single‐institution datasets, raising concerns about overfitting and poor generalizability to diverse patient populations and clinical settings [6].
4. Feature Importance Bias: Common measures for assessing feature importance (e.g., Gini importance) can be skewed, leading to biologically or clinically implausible interpretations [7].
5. Lack of Standardized Evaluation: The absence of consensus on evaluation metrics and reporting standards makes it difficult to compare models objectively and assess their readiness for clinical implementation.

These gaps highlight a disconnect between technical innovation and clinical practicality. Collectively, they suggest that further gains in clinical AI will be difficult to achieve through incremental algorithmic optimization alone, underscoring the need for principled methodological frameworks that explicitly address robustness, interpretability, and generalizability.

Importantly, these limitations become more evident when clinical AI is situated within the broader evolution of ensemble learning over the past two years. In non‐medical domains such as computer vision, automation, and intelligent optimization, hybrid and ensemble frameworks have increasingly progressed toward automated ensemble construction, sensitivity‐driven feature selection, and adaptive model updating [8]. These approaches are designed to dynamically adjust model composition or feature relevance in response to data perturbations or distributional shifts, thereby improving robustness under changing conditions.

By contrast, most clinical ensemble models remain manually designed and largely static once trained, with limited incorporation of adaptive or sensitivity‐aware mechanisms. This discrepancy reflects not a lack of technical sophistication but rather unresolved methodological tensions between automation, interpretability, and clinical accountability. Consequently, clinical AI continues to rely heavily on empirical model aggregation strategies that may achieve strong local performance yet struggle to ensure stability and transferability across heterogeneous patient populations.

## 3 Emerging Methodological Advances and the Uncaptured Translational Gap

Over the past two years, significant methodological progress has occurred in hybrid modeling across engineering and computational sciences. Automated machine learning (AutoML), neural architecture search (NAS), Bayesian hyperparameter optimization, and sensitivity‐driven feature governance have enabled the construction of self‐configuring ensemble systems capable of dynamically adapting model structures and weights to data characteristics. In these domains, hybrid systems are no longer manually assembled combinations of learners, but algorithmically optimized pipelines designed to minimize bias–variance trade‐offs in a data‐driven manner [9, 10].

In contrast, most medical hybrid ensemble models remain manually curated, with algorithm selection and ensemble weighting often determined heuristically. While such approaches may achieve high predictive accuracy in specific cohorts, they lack systematic optimization strategies and reproducibility across diverse clinical environments. This methodological asymmetry highlights a critical and under‐recognized gap: clinical AI has not yet fully integrated automated hybrid optimization frameworks that are already standard in other computational disciplines [11] resporeeesss.

Bridging this gap requires transitioning from static ensemble assembly toward automated, adaptive, and sensitivity‐aware hybrid architectures that can evolve alongside expanding clinical datasets.

## 4 A Principled Hybrid Ensemble Framework for Broad Clinical Utility

It is important to clarify the positioning of the proposed framework within the broader landscape of ensemble learning methodologies. Unlike fully automated or adaptive ensemble systems developed in other domains, which primarily pursue performance optimization through automation, the framework presented here is intentionally designed as a clinically feasible intermediate paradigm. Its primary objective is not maximal automation, but methodological robustness under real‐world clinical constraints, where interpretability, validation transparency, and regulatory accountability remain essential.

Accordingly, this framework emphasizes principled integration rather than algorithmic novelty. By aligning ensemble construction, validation philosophy, and explainability within a unified design paradigm, it functions as a translational scaffold that complements, rather than replaces, automated learning systems.

To address these challenges, we propose a methodological framework built on three foundational principles designed for cross‐disease applicability.

### 4.1 Principle 1: Balanced Integration Over Isolated Optimization

The goal is not to identify a single “best” algorithm but to construct ensemble paradigms that strategically integrate diverse learners. This integration aims to capitalize on their complementary strengths, thereby reducing the bias and variance inherent in any single model and improving generalization [12, 13, 14]. The selection process must be guided by the clinical context, data characteristics, and a clear objective to maintain a balance between predictive accuracy, model complexity, and interpretability.

### 4.2 Principle 2: Consensus‐Driven Validation

Robust clinical translation requires validation beyond a single dataset. We advocate for a consensus evaluation strategy that employs multiple, independent validation cohorts (e.g., geographically distinct or demographically varied populations). The final model is selected based on aggregate performance metrics (e.g., average concordance index) across these cohorts. This approach minimizes the risk of overfitting to local data artifacts, reduces selection bias, and provides a more realistic estimate of model performance in varied hypothetical clinical scenarios.

### 4.3 Principle 3: Explainability through Cooperative Game Theory

To tackle the “black box” problem and unbiasedly assess feature contribution, frameworks must incorporate advanced explainability techniques. We emphasize the integration of Shapley Additive exPlanations (SHAP), a method rooted in cooperative game theory. SHAP quantifies the average marginal contribution of each feature across all possible predictions, offering a unified, more reliable measure of importance compared to model‐specific metrics. This enhances transparency, facilitates biological interpretation, and helps clinicians understand and trust model outputs [15].

Nevertheless, explainability itself introduces methodological uncertainties that warrant explicit consideration. In high‐dimensional clinical datasets with correlated features, SHAP‐derived attributions may vary substantially under minor perturbations, and importance rankings may diverge from sensitivity‐based assessments. Such discrepancies indicate that feature attribution should not be interpreted as a stable or causal property, but rather as a robustness indicator that is contingent on data distribution and model structure.

Moreover, when ensemble predictions are aggregated across heterogeneous learners, feature contributions may reflect consensus effects rather than individual model behavior, potentially obscuring mechanistic interpretation. These considerations underscore the importance of interpreting explainability outputs in conjunction with sensitivity analysis and calibration, rather than in isolation.

## 5 Cross‐Disease Applicability and Methodological Synthesis

The strength of this principled approach lies in its generalizability. The same core challenges—high‐dimensional data, need for interpretability, and demand for robust validation—are encountered across specialties [16].

In Cardiovascular Medicine, consensus‐validated ensemble models can enhance perioperative risk stratification, helping to forecast complications more reliably across different populations and hospital settings [1].

In Oncology, such a framework can improve molecular subtype classification and prognosis prediction by integrating multi‐omics data through an ensemble lens while providing interpretable biomarkers [2, 17].

In Chronic Disease Management, where data may be limited or heterogeneous, the balanced and robust nature of this framework can yield more generalizable models for progression prediction or personalized intervention planning [18].

**FIGURE 1:**
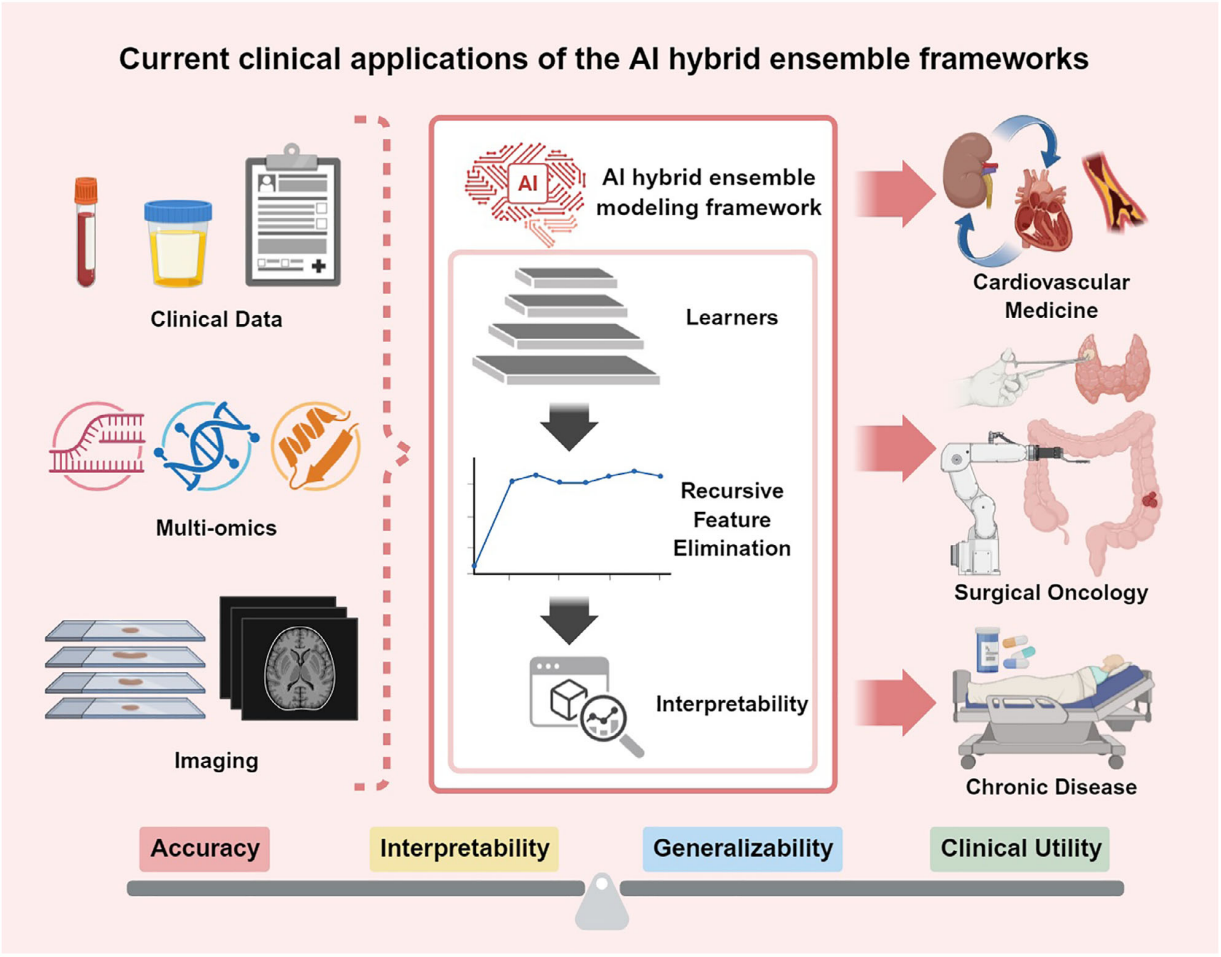
Schematic overview of the AI hybrid ensemble framework for clinical prediction. The left panel illustrates representative heterogeneous data inputs, including multi‐omics data (such as genomics and transcriptomics), routine clinical variables, and medical imaging modalities (e.g., CT and MRI), which collectively capture complementary biological and phenotypic information across diseases. The middle panel depicts the core AI hybrid ensemble framework, where multiple diverse learning algorithms are integrated to construct ensemble predictors, followed by consensus‐based evaluation across independent cohorts to enhance robustness and generalizability. Model‐agnostic explainability methods are incorporated to provide transparent feature attribution and improve interpretability. The right panel highlights downstream clinical applications, including risk stratification, prognosis prediction, and decision support across different disease contexts. The framework is intended as a generalizable methodological scaffold rather than a disease‐ or algorithm‐specific implementation.

As illustrated in Figure 1, our framework integrates multi‐omics data (e.g., genomics, transcriptomics) and medical imaging inputs (e.g., CT, MRI) into a hybrid ensemble pipeline, culminating in a consensus‐based predictive output. Thus, the framework serves as a methodological scaffold, adaptable to different data types (e.g., imaging, genomics, clinical variables) and clinical questions, moving the field toward reusable and reliable predictive modeling paradigms.

## 6 Technical Assumptions and Structural Limitations

Despite their promise, hybrid ensemble frameworks rely on several assumptions that warrant explicit acknowledgment.

First, ensemble integration does not guarantee improved generalization under substantial distributional shifts. Consensus validation reduces but does not eliminate bias introduced by unmeasured population heterogeneity. Second, explainability tools such as SHAP quantify conditional feature contributions rather than causal effects, and their stability may vary across resampling schemes. Third, automated optimization techniques may increase computational complexity and require infrastructural resources unavailable in lower‐resource clinical settings [19, 20].

Recognizing these structural constraints is essential to prevent overinterpretation and to promote responsible implementation of AI systems in high‐stakes medical environments.

## 7 Future Directions: Pathways to Translation and Collaboration

**FIGURE 2:**
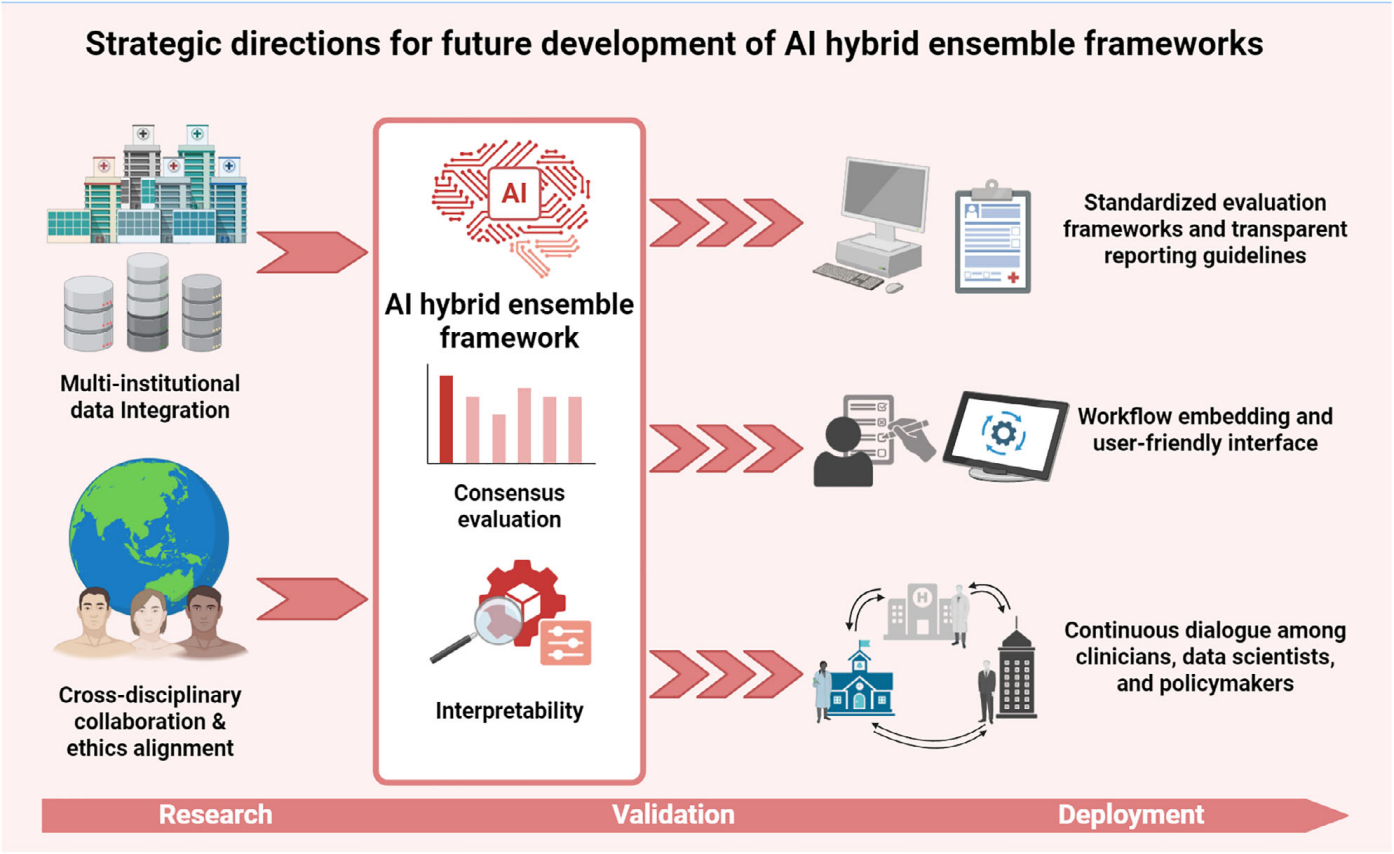
Conceptual roadmap for the future development and translation of AI hybrid ensemble frameworks. The figure outlines key pathways required to advance hybrid ensemble models from methodological concepts to clinically deployable tools. Core elements include multi‐institutional data collaboration to enhance population diversity, standardized evaluation and reporting frameworks to improve reproducibility and fairness, and interdisciplinary integration among clinicians, data scientists, and healthcare systems engineers. Additional considerations include clinician‐centered interface design, workflow compatibility, and resource‐aware implementation to facilitate equitable and sustainable adoption across healthcare settings.

As summarized in Figure 2, future development of AI hybrid ensemble frameworks will depend on coordinated progress in data sharing, standardization, and clinical integration. Several priorities will define the next stage of development. First, multi‐institutional and cross‐national collaborations are essential to build large, diverse, and representative datasets that reflect real‐world patient heterogeneity. Second, standardized evaluation frameworks and transparent reporting guidelines must be established to ensure fairness, reproducibility, and comparability across studies. Third, the engineering of clinician‐centric and user‐friendly AI platforms that seamlessly integrate with clinical workflows will allow clinicians to access advanced predictive insights without additional technical or cognitive burden. Finally, promoting continuous interdisciplinary dialogue among data scientists, clinicians, ethicists, and healthcare administrators is crucial to align technical development with practical clinical needs, ethical standards, and implementation pathways. Together, these efforts will pave the way for an ecosystem of AI‐enabled healthcare characterized by transparency, scalability, and clinical trustworthiness.

From a methodological standpoint, several actionable research directions emerge from this framework. First, future studies should systematically integrate sensitivity analysis with SHAP‐based explainability to evaluate the stability of feature importance under controlled perturbations, thereby distinguishing robust predictors from fragile artifacts. Second, sensitivity‐driven or robustness‐aware feature selection strategies represent a promising complement to ensemble learning, particularly in high‐dimensional clinical settings where feature correlations are common. Third, semi‐automated ensemble updating schemes that permit constrained model re‐weighting across cohorts—while preserving interpretability—may offer a practical balance between adaptability and clinical transparency.

Together, these directions outline concrete methodological pathways for empirically testing, refining, and extending hybrid ensemble frameworks beyond conceptual discussion.

## 8 Conclusion

This Perspective articulates a vision for clinical AI that prioritizes methodological principles for broad utility over narrow technical excellence. By advocating for hybrid ensemble frameworks grounded in balanced design, consensus validation, and rigorous explainability, we provide a roadmap for developing predictive tools that are not only accurate but also robust, interpretable, and trustworthy. Embracing such a principled approach is a critical step toward accelerating the responsible and effective integration of AI into routine clinical practice, ultimately improving patient care across a spectrum of diseases and healthcare settings.

## Author Contributions

H.Z., G.Z., X.H., C.Y., and R.W.: study design & manuscript writing. S.Z., Y.Y., Y.C., S.P., and G.Z.: study design & manuscript revision.

## Conflicts of Interest

The authors declare no conflicts of interest.

## Consent for Publication

All authors have read and approved the final version submitted.
